# Community knowledge and perceptions on malaria prevention and house screening in Nyabondo, Western Kenya

**DOI:** 10.1186/s12889-019-6723-3

**Published:** 2019-04-23

**Authors:** Peter Njoroge Ng’ang’a, James Mutunga, George Oliech, Clifford Maina Mutero

**Affiliations:** 1 International Centre of Insect Physiology and Ecology (ICIPE), P.O. Box 30772, Nairobi, Kenya; 2 School of Public Health, Jomo Kenyatta University of Agriculture and Technology, P.O. Box 62000, Nairobi, Kenya; 3 School of Health Systems and Public Health, University of Pretoria Institute for Sustainable Malaria Control (UP ISMC), University of Pretoria, Private Bag X363; Pretoria, Pretoria, 0001 South Africa; 4 Present Address: Department of Entomology, KEMRI/US Army Medical Research Directorate-Africa, P.O. Box 54-40100, Kisumu, Kenya

**Keywords:** Knowledge, Perception, Screening, Malaria, Mosquitoes, Nets

## Abstract

**Background:**

Screening of houses to prevent mosquito entry is increasingly being recommended as an effective and practical method against malaria transmission through reduced human-mosquito contact. The objective of the study was to assess community knowledge and perceptions on malaria prevention and house screening in a malaria endemic area of Western Kenya.

**Methods:**

A cross-sectional household survey was conducted in 2017 in Nyabondo area of western Kenya. A total of 80 households were randomly selected to participate in the study within 16 villages. Structured questionnaires, focus group discussions and key informant interviews were used to collect data.

**Results:**

A total of 80 respondents participated in the survey and more than half (53.8%) reported to have attained primary education. About 91% of the respondents had previously seen or heard malaria messages and this was associated with the respondents level of education (χ2 = 10.163; df 4; *P* = 0.038, 95% CI). However, other variables like gender, marital status, religion and occupation were not significantly associated with knowledge in malaria. Insecticide treated mosquito nets was by far the most reported known (97.4%) and applied (97.6%) personal protective while only 15.6% respondents were aware of house screening. The major reason given for screening doors, windows and eaves was to prevent entry of mosquito and other insects (> 85%). Lack of awareness was the major reason given for not screening houses. Grey colour was the most preferred choice for screen material (48%), and the main reason given was that grey matched the colour of the walls (21%) and did not ‘gather’ dust quickly.

**Conclusion:**

House screening was not a common intervention for self-protection against malaria vectors in the study area. There is need to advocate and promote house screening to increase community knowledge on this as an additional integrated vector management strategy for malaria control.

**Electronic supplementary material:**

The online version of this article (10.1186/s12889-019-6723-3) contains supplementary material, which is available to authorized users.

## Background

As of 2016, sub-Saharan Africa carried the vast majority of the global burden of malaria cases (90%) and deaths (92%); 70% of the deaths were in children under five [1]. Use of long-lasting insecticide-treated nets (LLINs) and indoor residual spraying (IRS) significantly contributed to a 60% decline in malaria mortality between 2000 and 2015 [1, 2]. However, the effectiveness of IRS and LLIN strategies is threatened by widespread development of insecticide resistance among vectors in most of the malaria-affected countries [3–5]. Consequently, the 2017 WHO report indicates that in the year 2016, an estimated 216 million cases of malaria were reported, which represents an increase of about 5 million cases over 2015 and that the deaths reached 445,000, a similar number to the previous year [3]. This demonstrates a stagnation of progress in the fight against malaria. For this reason, new mosquito control tools and strategies are needed to reinforce currently used methods in order to consolidate the gains made in the fight against malaria. In spite of current malaria vector control strategies focusing mostly on LLINs and IRS, a number of other measures can be implemented at household level to significantly reduce mosquito bites in humans. These strategies include integration and installation of house screening on doors, windows and eaves in order to prevent entry of adult mosquitoes in addition to environmental management aimed at eliminating mosquito breeding places near houses [6, 7]. Installation of house screening is necessary considering that more than 80% of malaria transmission occurs indoors, primarily at night [8]. This is, therefore, the most vulnerable time for people to be bitten and infected with malaria or other mosquito-borne pathogens. Thus, closing windows or screening of windows and open eaves can reduce the chances of mosquito bites, hence potentially lowering the occurrence of malaria, where mosquitoes usually feed on people indoors [6, 9, 10]. Recently, clinical trials have shown that both full house screening and ceilings provide valuable protection against anemia and exposure to malaria transmission in rural parts of The Gambia and Equatorial Guinea [6, 9, 11, 12]. Other studies in the Gambia, Guinea, Tanzania and Ethiopia have shown that house screening is a potential, sustainable and effective intervention in preventing mosquito entry into houses [5, 10, 12–16]. In related studies in Western Kenya, house modifications involving insect screen ceilings made from locally available materials and use of insecticide-treated eave tubes resulted in significant reductions in human exposure to malaria vectors representing the first and promising results with this novel control tool against malaria vectors [13, 17].

Improvement in housing design have contributed to reduction of malaria in many parts of the world [18, 19]. Preventing mosquitoes from entering houses has other additional advantages such as protecting all household members equally and at all times whilst indoors and offering protection against other vector borne diseases through integrated vector control [12].

While it might seem obvious that screening of houses can protect people against mosquito bites and malaria, this intervention is not common in many African rural communities [9, 11, 20]. The objective of the study was to determine the status of house screening in a community living in a malaria endemic area of western Kenya and to evaluate the community’s knowledge and perceptions on available interventions.

## Methods

### Study area

The study was carried out in Nyabondo Plateau, located in Upper Nyakach, 30 km on the North-Eastern part of Lake Victoria, in Kisumu County, Kenya. Nyabondo lies between an altitude of 1520 m and 1658 m above sea level, and 0° 23′ 0 S and 34° 58′ 60 E. The area is host to an estimated 34,000 people with a high population density of nearly 368 persons per square km. The main livelihood activities in the area include subsistence farming, mainly of maize, cassava and sweet potatoes, small scale livestock rearing and brick-making [21, 22]. The area receives two main rainy seasons i.e. the long rains (March to June) and short rains (September to November) with substantial annual variation. Agriculture is the key livelihood activity, employing 60% of the total population and supplying over 52% of household earnings. The community members in Upper Nyakach access health services from four health facilities including Nyabondo Hospital. There are several market centres, the main one being Sondu market. The main public health problems in the area are malaria and HIV/AIDS. Malaria is endemic in the Lake Victoria region, with a reported average prevalence of 27% in 2015 [22, 23]. The brick-making pits and poorly managed or abandoned fish ponds in Nyabondo area create ideal breeding sites for mosquitoes, thus contributing to malaria transmission in the area [21, 24, 25]. Previous entomological surveys in Nyabondo found that larval *Anopheles* mosquitoes bred in both temporary and permanent habitats with *An. arabiensis* being the main malaria vector species (99.3%), followed by *An. gambiae* (0.7%) [21, 25]. The overall poverty incidence in Nyabondo is approximately 61%, perpetuated by inadequate agricultural technology, poor roads, water and sanitation systems.

### Study design

The study was a cross-sectional ethnographic/phenomenology household survey conducted between December 2016 and January 2017 in Nyabondo, Western Kenya. The study area was clustered into 16 villages based on the existing administrative boundaries and roads. Five households were randomly selected from each village for inclusion in the study with an aim of assessing community knowledge and perceptions on malaria prevention and house screening.

### Data collection techniques

Structured questionnaires, key informant interviews and Focus Group Discussions [FGDs] were used to collect data from the respondents (Additional files 1 and 2). Pretesting of the questionnaires was done in a non-study village and adjustments were made accordingly. Informed verbal consent was obtained from the household heads and interviews using structured questionnaires were conducted among 80 randomly selected heads of households or their spouses. In cases where household heads were absent, the next responsible adult of 18 years and above from the same household was interviewed. Questionnaires were prepared in English and verbally translated into the local language (Luo) during face to face interview sessions. Questionnaires and FGD questions focused on various sub-themes including knowledge on malaria prevention, use of personal protection measures against malaria and mosquito bites, as well as perceived benefit for screening doors, windows, eaves, and attitude, perception & practice on house screening. FGD participants were purposefully selected while the discussion themes were derived in advance from both initial data and household questionnaire. Households questionnaire administration took around one hour while FGD interview took between 1 and 1.5 h. Further probing was done to gather more information, in total 34 Females and 11 Males participated in four different FGDs in the area. Since the FGD themes were not much gender sensitive and confidential, the participants were adults of 18 years and above, composed of both gender and different age groups.

### Data management and analysis

Data was entered in MS Excel, cleaned and checked for errors by an independent person. Corrections were made before being coded and processed using Statistical Package for Social Science (SPSS) version 21. Association between dependent and independent variables were cross tabulated and measured by use of Pearson Chi-Square Tests.

## Results

### Socio-demographic characteristic of respondents

#### Gender and main occupation

Out of the total respondents, 67.5% were females and 32.5% were males. The main reported occupation and economic activity in the area was farming (75%) followed by business (12.5%). [Table 1].

#### Highest level of education

More than half (53.8%) of the respondents had attained primary education, with 21.3 and 6.3% of the respondents reported to have completed secondary school and university/college education, respectively [Table 2].

### Housing design/characteristics

On assessing the respondent’s house design, 93.8% of the houses had their walls made up of mud and poles and 97.5% of the roofs were made up of iron sheets. Most of the houses had un-cemented floor (76.3%) and windows and eaves in the study area were open (95 and 97.5%, respectively). Kerosene lamp, firewood/charcoal were the main local sources of energy for lighting (62.5%) and cooking (97.8%) for the community. Several parts of the world use of firewood and charcoal has been attributed to reduced mosquito entry in huts [26, 27] and therefore information was sought for such practices in the study area There was no significant association between type of walls of the house and respondents socio-demographic characteristics like occupation (χ2 = 2.863; df 6; *P* = 0.826, 95% CI), religion and level of education (χ2 = 4.580; df 6; *P* = 0.599, 95% CI) and (χ2 = 10.591; df 8; *P* = 0.226, 95% CI) respectively.

**Table 1:** Main occupation of the respondents

Occupation	Frequency	Percent	Cumulative %
Business	10	12.5	12.5
Farmer	60	75	87.5
Salaried employed	2	2.5	90
Unemployed	8	10	100
Total	**80**	**100**

**Table 2:** Respondents highest level of education

Variable	Frequency	Percent	Cumulative %
Primary School (Completed)	23	28.8	28.8
Primary School (Not completed)	20	25	53.8
Secondary School (Completed)	17	21.3	75
Secondary School (Not Completed)	15	18.8	93.8
University/College	5	6.3	100
Total	**80**	**100**	

### Information sources on malaria prevention and control

**Fig. 1:**
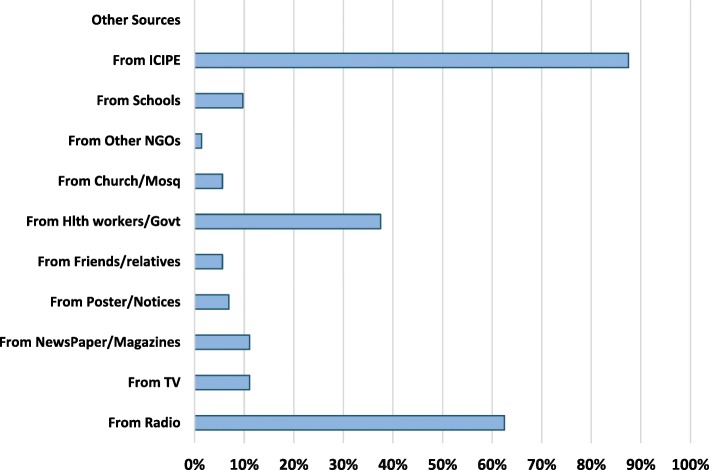
Sources of malaria prevention and controls messages (Multiple responses)

Ninety one percent (91.3%) of the respondents reported to have previously received, seen or heard malaria related messages in the area-in this text referred to as previous access to malaria prevention/control. Two leading reported source of malaria related information in the area was ICIPE (87.5%) and Radio (62.5%) [Fig. 1]. Government health department was mentioned by 37.5% of the respondents for its involvement in nets distribution and awareness creation through health education and promotion mainly by local community health volunteers [CHVs]. International Centre of Insect Physiology and Ecology [ICIPE] was highly mentioned due to its many years of involvement in mosquito larviciding activities, community mobilisation and awareness creation for malaria control in the study area. There was association between the number of respondents who reported to have seen or heard any malaria prevention/control information and the level of education (χ2 = 10.163; df 4; *P* = 0.038, 95% CI). All interviewed respondents who had high school education and above confirmed to have prior malaria prevention/control information. However, other socio-demographic variables like gender, marital status and occupation were not related. On malaria information and communication messages, use of mosquito nets was by far mentioned as the most recalled or widely known personal protection method reported in the area and reported by 94.7% of the respondents. Filling or Leveling of breeding sites and clearing household’s refuse or proper waste disposal were also the two most recalled environmental management messages at household level with 68 and 52% responses respectively [Table 3].

**Table 3:** Communicated malaria prevention and controls messages

Personal Protection method messages			
		Count	Column N %
1	Use Mosquito net-untreated	36	64.3%
2	Use Treated Mosquito net	17	30.4%
3	Use Insecticide spray	2	3.6%
4	Use Preventive medicine	18	32.1%
5	Use Screen windows/eaves/doors	0	0.0%
6	Use Light the fire/coils	0	0.0%
7	Use Mosquito skin repellents	2	3.6%
	Total	**56**	**100.0%**
Environmental management messages at household level			
1	Clearing HH Refuse/waste disposal	26	52.0%
2	Filling Breeding sites	34	68.0%
3	Clearing canals &Vegetation around houses	16	32.0%
4	Destroying water Receptacles	18	36.0%
5	Others Messages	1	2.0%
	Total	**50**	**100.0%**

### Awareness and application of malaria prevention and control methods

**Fig. 2:**
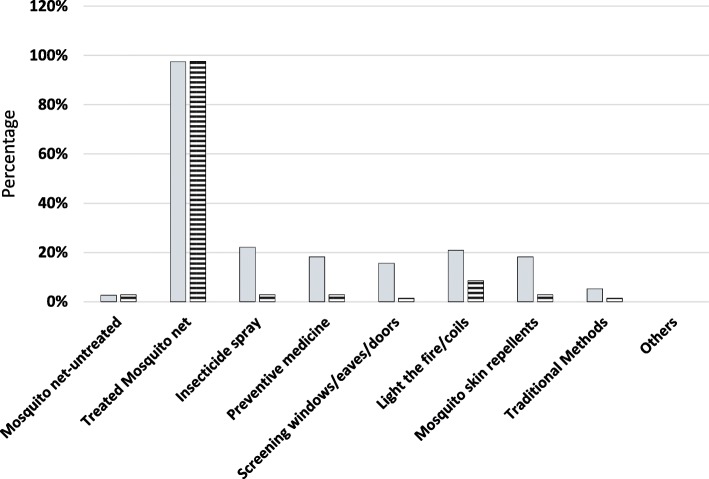
Known and used personal protection methods

**Fig. 3:**
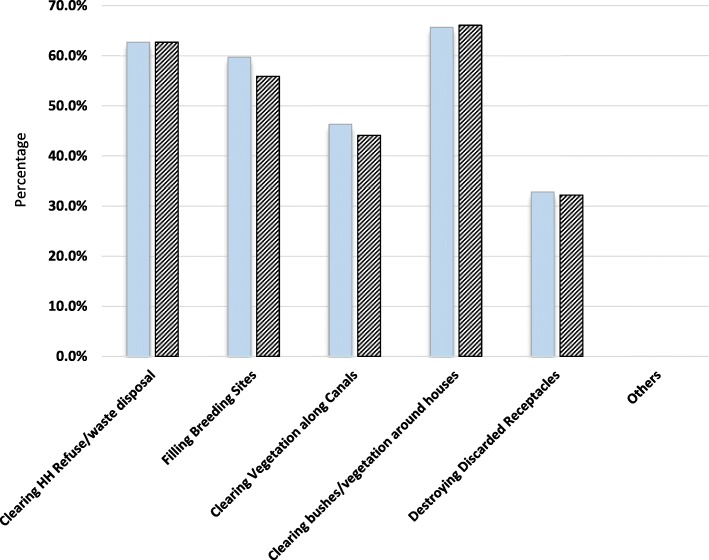
Known and applied environmental management methods

Insecticide treated mosquito nets was by far the most stated known (97.4%) and used (97.6%) personal protective method in the area [Fig. 2] and only 15.6% of the respondents were aware of screening of windows, eaves and doors. On environmental management practices, 65.7% of the respondents acknowledged clearing bushes/vegetation around houses in the community and 62.7% reported clearing household refuse/waste. About 60 and 46% reported filling breeding sites and clearing vegetation along canals respectively [Fig. 3]. There was high reported [recalled] application of environmental management practices at household level with 66.1% of the respondents reporting clearing bushes/vegetation around houses. Lighting of fire/coils was reported to be known (20.8%) and applied (8.5%) at household level and its use was associated with the respondents level of education (χ2 = 13.303; df 4; *P* = 0.010, 95% CI). Lighting of fire/coils and use of traditional methods were also expressed during FGD:

“Some people still use coils and shrubs (Lantana Kamara) to smoke houses even today, when the shrub is not properly managed in the compound it brings mosquitoes and if you burn it at night when it is green it repel mosquitoes, The methods are only effective when the smoke remains inside the house” (Nyamaroka FGD)

“Coils are effective but not 100% compared to nets, we use then when we spend our time outside at night during *‘matanga’* (night ceremonies conducted to mourn the dead). They cause *‘homa’* (sneezing) especially to people who are allergic” (Naki/Sigoti FGD).

### Perceived reasons for screening doors, windows and eaves

**Fig. 4:**
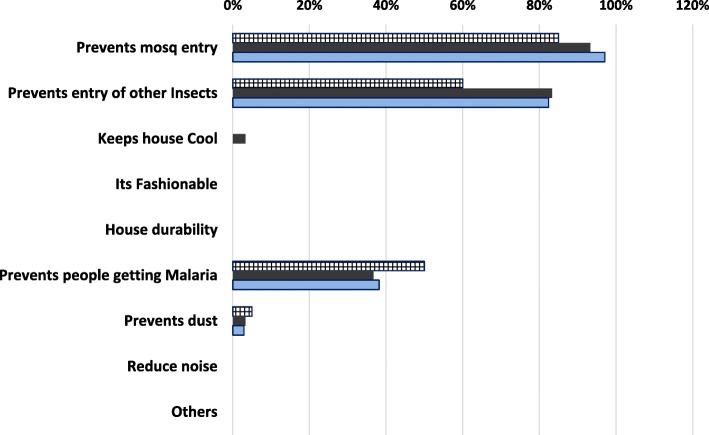
Perceived Reasons for screening doors, windows and eaves

About 58% of the respondents acknowledged not having heard any prior information or knowledge on house screening. Major perceived reasons given for screening doors, windows and eaves were to prevent entry of mosquito and other insects into houses (> 85%), with considerable number of respondents mentioning preventing people from contracting malaria [Fig. 4]. In one of the FDGs, an elderly male participant explained how in the early day’s house screening benefited the community.

“During our early days people used to screen houses locally by closing the eaves, we used to stay by the lake side (Lake Victoria) and the malaria was minimal, there were no mosquitoes and malaria during that time”.

There was no association between reason given for house screening and sociodemographic characteristics of the respondent like the level of education, occupation, religion, gender and village of origin.

### Reasons for not screening houses

Lack of knowledge on how to screen houses (78%) was one of the major reasons given for not screening doors, windows and eaves. The second major reason given was economic/affordability factors at household level, i.e. 43% for doors, windows and 44% for the eaves [Table 4]. Similar reasons were also captured from FGD where participants were willing to participate in house screening (i.e. screen houses on their own) if given information on how to screen.

“Here people don’t have knowledge or information on house screening and that is the reason we don’t screen. If given opportunity, local people will be much willing to participate (Actively & passively) and contribute to malaria control and house screening”. “Also ignorance, negligence, and carelessness are hampering use of most personal protection methods in the community. We can network and collaborate at all times, we can contribute by cost sharing in order to fight malaria in the community” (Siatok windows FGD).

“Nowadays we don’t screen our houses because we have nets, we feel that nets give us protection. If we don’t screen, air flows well within the house” (Nyamaroka FGD).

The average cost of eave-screening one house in the area was assessed and estimated at 30 US$ (cost for local screening material, nails, wood and labor) and affordability was mentioned as the second most important factor.

**Table 4:** Major reasons for not screening houses

Variable	Reasons for not Screening:					
	Doors		Windows		Eaves	
	Count	Column %	Count	Column N %	Count	Column N %
Not effective	1	2%	1	2%	1	1%
Hot at night	0	0%	0	0%	0	0%
Prevents ventilation	0	0%	0	0%	0	0%
House do not look good	0	0%	0	0%	0	0%
Cultural reason	1	2%	1	2%	2	3%
Washing/maintaining hard	0	0%	0	0%	0	0%
House design problems	0	0%	0	0%	1	1%
Economic/affordability	29	43%	29	43%	31	44%
Do not Know to do it	53	78%	53	78%	55	78%
Lack of time	0	0%	0	0%	0	0%
Others reasons	1	2%	1	2%	1	1%
Total	68	100%	68	100%	71	100%

There was an association between the respondents previous knowledge of malaria prevention/control and the reasons given for not screening doors (χ2 = 13.006; df 5; *P* = 0.016, 95% CI) windows (χ2 = 13.006; df 5; P = 0.016, 95% CI) and eaves (χ2 = 14.481; df 6; *P* = 0.025, 95% CI).

### Colour preference for screen materials

**Fig. 5:**
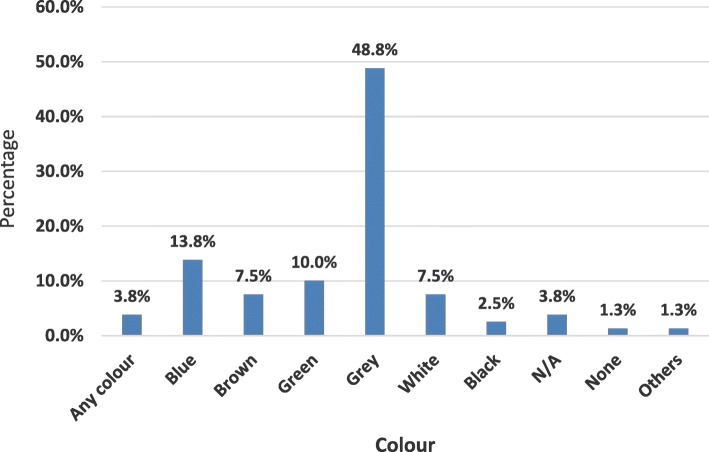
Preferred colour for screen material

Respondents from different households were shown various coloured samples of screening materials (white, black, blue and grey) and contrasted against the eaves area of their houses to assess their preferred colours. Grey was the most preferred colour (48.8%), followed by Blue (13.8%) and green (10%) [Fig. 5]. The Grey colour was preferred because it was reported to match with the walls of the house (21%) and didn’t display or “gather” dust quickly (16.3%). Same reasons were given during various FGDs conduced in the study area. There was no significant variation between the colour preference for the eaves material and the walls of the house. When asked about their willingness to screen their house, all the respondents who participated in this survey were willing to screen their houses if given information on how to screen. When asked about IVM participation, 96.3% of the respondents were willing to passively and actively participate and collaborate in future IVM activities in the community with an aim of preventing/controlling malaria transmission in the area.

## Discussion

Nyabondo is one of the malaria endemic areas in western Kenya [28, 29]. At the time of the study, malaria was reported to be one of the leading causes of morbidity and mortality in the study area with a reported 92 students from a nearby girl’s boarding school being hospitalised in a local mission hospital presenting with malaria signs and symptoms (local dispensary records). The morbidity was attributed to lack of appropriate personal protection measures in most boarding schools at night and also probably due to migration of students from areas where malaria is not endemic.

In sub-Saharan Africa, 80% of malaria transmission occurs indoors primarily at night, with dusk through dawn being the most vulnerable times for humans to be bitten by mosquitoes [8]. In one of the FGDs it was noted that local people are knowledgeable about the peak biting time of the local vectors as confirmed from one of the focus group discussions (Siatok widows) where participants reported that female anopheles mosquitoes normally bite from 10 PM up to around 4 AM in the early morning. Field studies have shown that various species of *Anopheles* have become more efficient vectors and have developed behaviours that allow them to adapt to indoor feeding, targeting humans in homes and other built environments before dusk [8, 30–32]. *Anopheles gambiae s.l.*, a common and dominant species in the study area is able to find openings in the dwelling, such as windows, cracks, or open eaves, while outdoor feeders have not adapted this behavior [33, 34]. This vector species behaviour corresponds with the common characteristic feature of houses in the study area where 93.8% have walls made of mud & poles. Over 95% of the windows and eaves were unscreened thereby increasing the risk for house-entry by malaria vectors and that of other insect vectors. A modern, well-built housing can protect residents in many tropical countries by reducing house entry by mosquito vectors [19, 33–36]. In the Gambia, houses with walls made of mud blocks showed increased number of indoors mosquitoes as opposed to concrete, this was most likely due to presence of more cracks and aging of mud blocks [9, 11]. Open eaves were shown to be the main routes of entry by *An. gambiae s.l* which is also the main mosquito species in the study area where almost all windows and eaves were unscreened.

Previous studies have shown that, for interventions to be effective in reducing malaria morbidity they need to be based on specific risk factors that contribute to disease transmission [10]. These risk factors for malaria transmission include but not limited to environmental parameters like topography, proximity of dwelling places to mosquito breeding sites, house design, density of human populations, knowledge of local vector and use of vector control as well as their socio-economic status [15, 37]. All these were potential risk factor in the study area, which is host to an estimated 34,000 people with a high population density of nearly 368 persons per square km., characterized by a host of various economic activities like brick-making with hot spots of active abandoned brick making pits and poorly managed or abandoned fish ponds not far from dwelling places [21]. In other related studies in Africa malaria risk and transmissions has been shown to be strongly influenced by socio-demographic factors [9, 11]. In Nyabondo, 75% of the respondents were subsistence farmers and more than half reported to have low level of education (53.8%) during this study.

On knowledge and use of malaria vector prevention measures, majority of respondents (97.4%) reported being aware of use of treated mosquito nets while less than 22% of the respondents reported other personal protection including use of fire and coils [26, 27, 38]. This was also observed when respondents were interviewed on application of the same methods at household level. Despite most respondents confirmed use of treated nets, other personal protection methods were far less reportedly mentioned. In addition to lack of awareness or information on use of personal protection methods and socio-economic factors, participants in one of the FGDs mentioned ignorance and negligence as other reasons hampering both use of other personal protection methods and eaves screening at household level as shown in Fig. 2. To address this, there is need for regular community advocacy, communication, mobilization, and social promotion of available personal protection methods in the community through development of socially accepted Information, Education and Communication (IEC/BCC) strategies [39, 40]. The approach should focus on awareness creation, education and enabling local people to optimize use of available vector control options and enhance use of house screening in the community. This could be done through use of appropriate and suitable user-friendly approaches such as interpersonal communication, mass media, schools, mobile teams, community field days or public forums. Such approaches should be used to create awareness through drama, health dialogue, role plays and songs, during annual malaria and mosquito day’s activities in the area prior to onset of heavy rains and peak malaria transmission seasons.

Comparatively, there was relatively high awareness and use of environmental management practices at household level. This was attributed to dissemination of information by three main sources i.e. ICIPE (87.5%) radio (62.5%) and ministry of health CHVs (37.5%). However, environmental management interventions are normally not very effective on their own unless appropriately integrated with other control measures [41]. They do not have immediate effect in reducing the number of biting vectors and may take longer before reduction in vector population can be achieved. During FGDs, it was evident that local people rarely and actively participate in environmental management at village level unless there is a motivating factor to bring them together. This being common in most African rural settings, it is prudent for the community to understand both the vector ecology and the accrued short and long term benefits of environmental management [39, 42, 43]. During the FGDs participants confirmed that they understood the direct benefits of participating in environmental management and if given opportunity, they were much willing to participate in malaria control and house screening.

However, 57.5% of the respondents acknowledged not to have heard any prior information on house screening. Major perceived reasons given for screening doors, windows and eaves were to prevent entry of mosquito and other insects into houses with considerable number of respondents mentioning preventing people from contracting malaria. Understanding community knowledge and perception on house screening and the changing self-protection behaviors and the interactions of humans is essential in ensuring success and sustainability of community based vector control interventions in Africa. Despite house screening being not common in Nyabondo, large number of respondents perceived that prevention of mosquito entry and other insects is the main reason for house screening doors, windows and eaves (> 85%). This indicates that the community may need to be informed on the need to protect their household against mosquito bites through house screening which is expected to significantly translate into preventing residents from contracting malaria and also the overall social and economic benefits accrued at household level. In related studies in Dar es Salaam, Tanzania, similar results were observed where many respondents understood that installation of ceilings protects them from mosquitoes and some respondents associate this with protection against malaria infection [15, 36]. This indicates that, considering other socioeconomic determinants, house screening can be easily promoted based on its multiple benefits particularly in rural areas.

Another potential advantage of house screening is the equity with which it protects all members of the household at all times while indoors unlike LLINs which primarily give protection to those with a net during sleeping hours only [44]. It can offer protection from other vector borne diseases like Lymphatic Filariasis, Rift Valley Fever, O’Nyong Nyong, Kala-azar, Dengue, Zika virus as well as malaria [13]. Perhaps the greatest benefit to house eaves screening would be its potential for integration with other vector control interventions including environmental management. Lack of knowledge on how to screen houses (78%) was one of the major reasons given for not screening doors, windows and eaves in the study area. The second major reason reported was economic/affordability factors at household level. Affordability of screening was rated at nearly 40% which implies that cost is an important consideration for this technology and ultimate assimilation by communities will depend on the netting material and user-care dependent durability of the screens. On screening materials colour preference, grey was the most preferred colour and the reasons given during FGD was that most houses in the area are semi-permanent and are constructed using locally available materials except for the roofing, therefore the colour matched with the walls of the house and it didn’t display or get dirty quickly/easily. All questionnaire respondents confirmed that they were willing to screen their houses if given information and skills. House screening has also been shown to have high levels of acceptability in Africa when properly implemented, which could also make it an effective intervention in areas with high malaria transmissions [20, 37, 41].

## Conclusion

The study demonstrated that house screening was not a common intervention against malaria vectors in the area. There is need for advocacy, communication, mobilization, and social promotion of house screening and integrating it with other available vector control interventions.

## Additional files

Additional file 1: House Screening Questionnaire. Household questionnaire. Screening Questionnaire (PDF 707 kb)

Additional file 2: FGD Interview Guide. FGDs Guide. (PDF 113 kb)

## Abbreviations

BCC: Behaviour Change Communication
CHVs: Community Health Volunteers
DDT: dichlorodiphenyltrichloroethane
FGD: Focus Group Discussion
IEC: Information, Education and Communication
IRS: Indoor residual spraying
IVM: integrated vector management
KEMRI: Kenya Medical Research Institute
LLINs: Long lasting insecticide-treated nets
RBM: Roll Back Malaria Partnership
SDGs: Sustainable Development Goals
SERU: Scientific and Ethic Review Unit
SPSS: Statistical Package for Social Science
