# Supplementary material for: Community knowledge and perceptions on malaria prevention and house screening in Nyabondo, Western Kenya
Source: BMC Public Health. 2019 Apr 23;19:423. doi: 10.1186/s12889-019-6723-3 (PMC6480882; doi:10.1186/s12889-019-6723-3)
Supplement: Supplementary file 1 — House Screening Questionnaire. Household questionnaire. Screening Questionnaire (PDF 707 kb) [file 12889_2019_6723_MOESM1_ESM.pdf]

HOUSEHOLD QUESTIONNAIRE ON MALARIA PREVENTION AND HOUSE SCREENING IN NYABONDO KENYA.

INTRODUCTION

"Hello, my name is ………………..from………….. I/we are carrying out a survey on malaria control in this area. I hope you can help us by answering some questions. Your participation will enable us to better understand the malaria situation and ways of controlling it in this area. I hope that you will feel free to spare some few minutes and discuss with me about malaria and other house screening issues that involve your household. You are not under obligation to participate in the study, but it is my desire that you do so. Do you agree?

Yes_____________ No_____________

(If yes, thank the respondent and briefly explain the objective of the study and confidentiality of the information).

NB: In the event that information is not made available after repeated visits please fill the box below and return the questionnaire.

SECTION 1: IDENTIFICATION

Interviewer Name…………………………………………………………Date……………………………

Village Name /Number………………………………………Total Occupants…………Under 5……

Number of Rooms (Main House) ……………….

SECTION 2: SOCIO-DEMOGRAPHIC PROFILE OF RESPONDENT (Tick Accordingly)

1. Relationship of the respondent to Head of Household

1	Head	
2	Wife	
3	Husband	
4	Son (above 18)	
5	Daughter (above 18)	
6	Others (Specify)	

2. Sex of respondent

Male		Female	

3. Highest level of education attained

1	Primary school (Not completed)	
2	Primary school (Completed)	
3	Secondary school (Not completed)	
4	Secondary school (Completed)	
5	University/ college	
6	Informal	
7	None	

4. Marital status

1	Married	
2	Single	
3	Separated	
4	Widowed	
5	Divorced	
6	Others (Specify)	

5. Religion

1	Catholics	
2	Protestants	
3	SDA	
4	Islam	
5	Others	

6. Main Occupation/source of Income

1	Farmer	
2	Business	
3	Salaried Employment	
4	Unemployed	
5	Others (specify)	

SECTION 3: HOUSE DESIGN AND CONSTRUCTION OF THE MAIN HOUSE (Tick Accordingly)

**7. WALLS:**		***Tick***	**8. ROOF**		***Tick***	**9. FLOOR:**		***Tick***
1	Grass & poles		1	Grass		1	Not cemented	
2	Mud & poles		2	Banana leaves		2	Cemented	
3	Mud bricks		3	Iron sheets		3	Others (Specify)	
4	Cement/ stone		4	Tiles/ Asbestos		4		
5	Iron sheets		5	Others (specify)				
6	Timber							
7	Others (Specify)							
**10. WINDOWS:**		***Tick***	**11. EAVES:**		***Tick***	**12. LIGHTING:**		***Tick***
1	Screened		1	Screened		1	Electricity/Solar	
2	Unscreened		2	Unscreened		2	Kerosene lamp	
3	No windows		3	No eaves		3	Firewood	
						4	No source of lighting	
**13. COOKING:**			**14**	No. of Windows				
1	Electricity/ solar			…………				
2	Kerosene			No. of Doors				
3	Firewood/ charcoal			………				
4	Gas							
5	Others (Specify)							

KNOWLEDGE ON MALARIA PREVENTION AND CONTROL:

15. Have you seen or heard any malaria prevention and control message from any source BEFORE?

1. Yes ☐ 2.No ☐

16. If Yes, Where did you see or hear these prevention and control message from? *(Multiple responses)*

	Source	Tick	What was the message? ***(write down the message)***
1	Radio		
2	T.V.		
3	Newspapers/magazines		
4	Posters / notices		
5	Friends/ relatives		
6	Health workers/Gov't officials		
7	Church / Mosque		
8	Other NGOs (Name them)		
10	School		
11	ICIPE Team		
11	Others (specify)		

17. What are some of the ways of controlling or preventing your household/family from malaria/Mosquito bite? (*Tick accordingly as they are mentioned*).

	Method	Methods Known to you *(tick)*	Methods Currently Applying *(tick)*
1	Use of mosquito net-untreated		
2	Use of treated mosquito net		
3	Use of insecticide spray		
4	Taking preventive medicine		
5	Screen windows, eaves, ceilings and doors		
6	Light the fire/coils		
7	Apply mosquito repellents to the skin		
8	Use traditional Methods (Specify)		
9	**Environmental Management (Household level):**		
	Clearing HH refuse/proper waste disposal		
	Filling/Levelling breeding sites		
	Clearing vegetation in canals		
	Clearing bushes/vegetation around houses		
	Destroying discarded receptacles		
	Others (Specify)		

ATTITUDE /PERCEPTION & PRACTICE ON HOUSE SCREENING

18. Do you have any information/ knowledge on house screening?

1. Yes ☐

2.No ☐

19. If yes, what are the Reasons for house

screening Doors, Windows and Eaves? (*Tick accordingly*)

S/N	Reason House Screening	Doors	Windows	Eaves
1	Prevents entry of mosquitoes			
2	Prevents entry of other insects			
3	Keeps house cool			
4	As a fashion			
5	House durability			
6	Prevents people from contacting Malaria			
7	Prevent dusts			
8	Reduce noise from outside			
9	Any other (Specify)			

20. Problems/reasons that prevent household owners not screen their houses in this area

S/N	Problem associated with house screening	Doors	Windows	Eaves
1	Not effective			
2	Too hot at night			
3	Prevents air ventilations			
4	House doesn't look good			
5	Not culturally accepted			
6	Washing/maintaining them is hard			
7	Housing designs problems			
8	Economic/affordability reasons			
9	Don't know how to do it			
10	Don't have time to apply			
11	Any other (Specify)			

21. Which Color of the screen would you prefer for your house and why?

	Colour Preference	*Tick*	*Reason*
1	White		
2	Blue		
3	Brown		
4	Green		
5	Grey		
6	Others (Specify)		

22. If given the information on how to screen would you be willing to do it? 1. Yes ☐ 2.No ☐

23. If NO, why? …………………………………………………………………………………………………

24. Are you willing to participate in other IVM activities other than house screening in this area?

	Willingness	*Tick*	*Reason*
1	YES		
2	NO		
3	Don’t Know		
