# Supplementary material for: Community knowledge and perceptions on malaria prevention and house screening in Nyabondo, Western Kenya
Source: BMC Public Health. 2019 Apr 23;19:423. doi: 10.1186/s12889-019-6723-3 (PMC6480882; doi:10.1186/s12889-019-6723-3)
Supplement: Supplementary file 2 — FGD Interview Guide. FGDs Guide. (PDF 113 kb) [file 12889_2019_6723_MOESM2_ESM.pdf]

FOCUS GROUP DISCUSSIONS GUIDE

Introduction

"Hello, my name is ……………..from …….... I/we are carrying out a survey on malaria control in this community. I hope you can help us by participating in some focused discussions on selected topics. Your participation will enable us to better understand vector control, malaria situation and ways of controlling it in this area. I hope that you will feel free to participate and discuss with me about malaria and its control in your village. You are not under obligation to participate in the discussion, but it is my desire that you do so.

SECTION A: BEDNET USE & HOUSE SCREENING

1. (a) Malaria is one of the the most frequently occurring disease in this area?
   (b) What are the main causes of malaria in this area and What activities contribute to this?

2. (a) Are mosquitoes a problem in this area? If Yes, How?
   (b) How do people get exposed to mosquitoe bites in this area?
   (c) What are the available personal protection measures against malaria & mosquito bites
   (d) What are peoples perception on their effectiveness (2c above)?
   (e) Do people use these methods and if not What hampers their use in the community?.

3. (a) Which categories /groups of people are most affected (at higher risk) by malaria in this area? (b) And what makes them to be at a higher risk ?

4. (a) Do people always sleep under insceticide treted net in this area? If Not, Why?

   (b) How are most of the nets acquired, their colour, the community preferred Colours, Shape and why?

   (c) When are bednets mostly used time (eg. Time of the days/days/ seasons/years)

   (d) What do local people think are the benefits of sleeping under a treated bednet

5 (a) Wher do local people get information on malaria prevention and controls?

   (b) If yes, What messages do they get?

6. (a) Do the local peolpe participate in environmental management aimed at vector control both at individual and community level and How? If no Why?

   (b) If any what are the advantages and disadvantages of community participation in vector control in this area?

7 (a) Do the local peolpe have any prior information or knowledge on house screening

   (b) If any how do they undestand it and major perceived reasons given for house screening doors, windows and eaves

8. (a) What are the major reason that makes local people not screening the doors, windows and eaves ?

   (b) In our previous household survey, local people preferred Grey colour (48.8%), & Blue (13.8%) for house screening materials, do you have any reason for preferring these colours ?

   (c) If given an opportunity, are the local people willing to participate in malaria control and screening of their houses ?

End of the discussion.

Thank all the participants for their contribution and spending their precious time with you.
